# Recurrence Rate during 5-Year Period after Suspension of Anti-Vascular Endothelial Growth Factor Treatment for Neovascular Age-Related Macular Degeneration

**DOI:** 10.3390/jcm13154317

**Published:** 2024-07-24

**Authors:** Shinichiro Chujo, Hisashi Matsubara, Yoko Mase, Kumiko Kato, Mineo Kondo

**Affiliations:** Department of Ophthalmology, Mie University Graduate School of Medicine 2-174 Edobashi, Tsu 514-8507, Japan; hmatsu@clin.medic.mie-u.ac.jp (H.M.); yokosun9@gmail.com (Y.M.); cqw14171jp@yahoo.co.jp (K.K.); mineo@med.mie-u.ac.jp (M.K.)

**Keywords:** age-related macular degeneration, AMD, anti-VEGF agent, intravitreal injection, treatment suspension, recurrence rate

## Abstract

**Purpose:** To determine the recurrence rate of neovascular age-related macular degeneration (nAMD) during a 5-year period after the suspension of anti-vascular endothelial growth factor (anti-VEGF) treatments. **Methods:** Thirty-four eyes of 34 nAMD patients who met the inclusion criteria and were treated by anti-VEGF drugs were studied. All met the treatment suspension criteria and were followed for 5 years after the suspension of the anti-VEGF treatment. Patients with a recurrence within one year were placed in Group A, and patients with a recurrence between 1 and 5 years were placed in Group B. The rate and time of a recurrence were analyzed using the Kaplan–Meier method. We also examined whether there were differences in the baseline factors of age, sex, subtype, treatment period, and treatment interval between Groups A and B. **Results:** Twenty-five of 34 eyes (73.5%) had a recurrence within 5 years of stopping the anti-VEGF treatments. Thirteen (52.0%) of the 25 eyes had a recurrence within 1 year, 4 (16.0%) eyes between 1 and 2 years, 4 (16.0%) eyes between 2 and 3 years, 2 (8%) between 3 and 4 years, and 2 eyes (8%) between 4 and 5 years. The baseline factors were not significantly different between Groups A and B. **Conclusions:** The results showed that the recurrence rate was highest within one year after the suspension of the anti-VEGF treatments, with a number of recurrences one year after the suspension. Clinicians should remember that nAMD may recur several years after the suspension of anti-VEGF treatments.

## 1. Introduction

Neovascular age-related macular degeneration (nAMD) is a major retinal disorder that can progress to a significant reduction in the visual acuity. This is important because the prevalence of nAMD is increasing in Japan and worldwide [1,2,3]. Anti-vascular endothelial growth factor (anti-VEGF) agents have been widely used to treat nAMD, and it has become the first-line treatment for nAMD [4]. The treat and extend (TAE) regimen in which the treatment interval is changed depending on the status of the retinal lesions, is widely used as the treatment regimen [5]. It is used because its proactive treatment features reduce the number of injections and hospital visits while maintaining as good a vision more than the As Needed Regimen [5,6].

Despite the benefits of the TAE regimen, the burden associated with long-term anti-VEGF treatments must also be considered. The burden associated with the treatments is not only the frequent hospital visits but also the financial and emotional burdens [7,8]. In addition to these difficulties associated with frequent treatments, the risks of complications associated with intravitreal injections must be considered [9,10,11,12,13].

Thus, to reduce these risks and burdens, it was proposed to suspend anti-VEGF treatments once the disease activity had stabilized [14]. However, the rate of recurrence after suspension is a concern [15]. Thus, Nguyen et al. reported that the best-corrected visual acuity (BCVA) was lower after a recurrence than at the time of the suspension [16]. Thus, the recurrence of exudations after suspension of treatment is an important factor to consider.

In contrast to trial data analysis, real-life data can be obtained from a group of patients who have been treated for various periods of time and have different treatment histories. Therefore, real-life data can be more consistent with that of actual clinical practice.

In an earlier study, our group studied the recurrence rates and other adverse events in nAMD patients two years after the suspension of their anti-VEGF treatments. The results showed that about 59% of patients had a recurrence within 1 year after the suspension of the anti-VEGF treatments. However, we did not have data on how the patients progressed after two years, i.e., the recurrence rates after 2 years. Thus, the purpose of this study was to determine the recurrence rates during a 5-year observation period after the suspension of treatment. In addition, we also examined whether an early resumption of treatment after a recurrence will maintain the visual acuity by analyzing the changes in the visual acuity after resumption of treatment in patients who had a recurrence within one year.

## 2. Materials and Methods

### 2.1. Study Design

The procedures used in this study were approved by the Ethics Committee of Mie University Hospital (approval number: H2021-088, UMIN000044144), and they conformed to the tenets of the Declaration of Helsinki. The medical records of patients diagnosed with nAMD between April 2009 and December 2023 who were treated with intravitreal injections of ranibizumab (Lucentis; Novartis, Brach, Switzerland) or aflibercept (Eylea; Bayer, Basel, Switzerland) at the Mie University Hospital (Mie, Japan) were analyzed.

### 2.2. Subjects

The inclusion and exclusion criteria were the same as those used in our earlier study [17]. Patients were included if they were >50 years of age, had been diagnosed and treated by intravitreal injections of ranibizumab or aflibercept for more than 2 years at the Mie University Hospital, were treated with the TAE regimen or the fixed dosing regimen, met the suspension of treatment criteria, and agreed to treatment suspension. Importantly, all of the patients had to have been followed for at least 5 years after the suspension of treatment.

The suspension criteria were an absence of exudations or hemorrhages for at least 48 weeks with continuous anti-VEGF treatment every 12 to 16 weeks. Patients were excluded if they had received photodynamic therapy, laser photocoagulation in the macular region, prior vitrectomy, intravitreal injection of steroids in the targeted eye, or had been treated in both eyes by an anti-VEGF agent during the study period for any type of retinal disease.

If a recurrence occurred within one year after the suspension of the anti-VEGF treatments, the patient was placed in Group A, and if a recurrence occurred one year after the suspension of anti-VEGF treatments, the patient was placed in Group B.

After meeting the conditions for suspension, the patient was presented with the option to suspend or continue the treatment, and the suspension was made at the patient’s request.

### 2.3. Post-Suspension Follow-Up Methods and Treatment Regimens

The post-suspension follow-up methods and treatment regimens were the same as those used in our previous study [17]. After the suspension of the anti-VEGF treatments, the condition of the lesions was determined by optical coherence tomography (OCT), fundus examinations, and the best-corrected visual acuity (BCVA) at each visit. The first visit after the suspension was at 3 months, and the subsequent visits were every 1 to 2 months at our hospital or at a clinic close to the residence of the patient during the first year after the suspension. During the second year, the visit interval was selected by the treating ophthalmologist, and the patients were examined every 2 to 4 months. This interval was continued until a recurrence occurred. In all eyes in which the anti-VEGF treatment was resumed, the presence of exudation was determined 1 month after the resumption. The treatment regimen used after the resumption was selected by the treating ophthalmologist.

### 2.4. Data Acquisition

The beginning of the treatment suspension was defined as the visit when the last anti-VEGF injection was given before the suspension criterion period (baseline). The recurrence point was defined as the visit when subretinal fluid (SRF), intraretinal fluid (IRF), or retinal hemorrhage was detected by OCT or fundus examinations. The demographic and clinical features extracted from the medical records included age, sex, past treatment times (months), number of injections, and subtype of AMD, viz. Type 1 or Type 2, or polypoidal choroidal vasculopathy (PCV). The decimal BCVA was measured in all eyes at each visit. The pretreatment subtype of AMD was determined from the fluorescein and indocyanine green angiographic images.

To investigate the differences in the clinical characteristics between Group A and Group B, we compared the disease type, age, duration of the treatment, and interval between the treatments in the two groups statistically.

As a secondary evaluation, we compared the changes in the BCVA at the time of resumption of treatment in Group A patients who had a recurrence within one year. The final period was the last examination, 5 years after the suspension of treatment. The examination times were then set at one-year intervals from the time treatment was suspended (1, 2, 3, 4, and 5 years).

### 2.5. Statistical Analyses

The descriptive data are presented as numbers, percentages, and medians with the first and third quartiles (Q1, Q3) and the 95% confidence intervals (CIs). The rate and recurrence times were analyzed using the Kaplan–Meier method. The Cox proportional hazard regression analysis was performed to estimate the hazard ratio for the recurrences by univariate and multivariate analysis of the age, sex, subtype of AMD, and past treatment period prior to the suspension. The selection of prior variables was based on the data in past publications [13,14], the results of univariate analyses, and clinical perspectives. For statistical analyses, Mann–Whitney’s U test, Fisher’s exact tests, Wilcoxon matched pairs signed-rank tests, and Friedman tests with post hoc Dunn tests were used. The decimal BCVA was converted to the logarithm of the minimum angle of resolution (log MAR) units for the statistical analyses. Tukey’s multiple comparison test was used to examine visual acuity trends within Group A. All *p* values were two-sided, and *p* < 0.05 was taken to be statistically significant. All statistical analyses were performed using R version 2.9.0.

## 3. Results

### 3.1. Selection of the Participants

**Figure 1:**
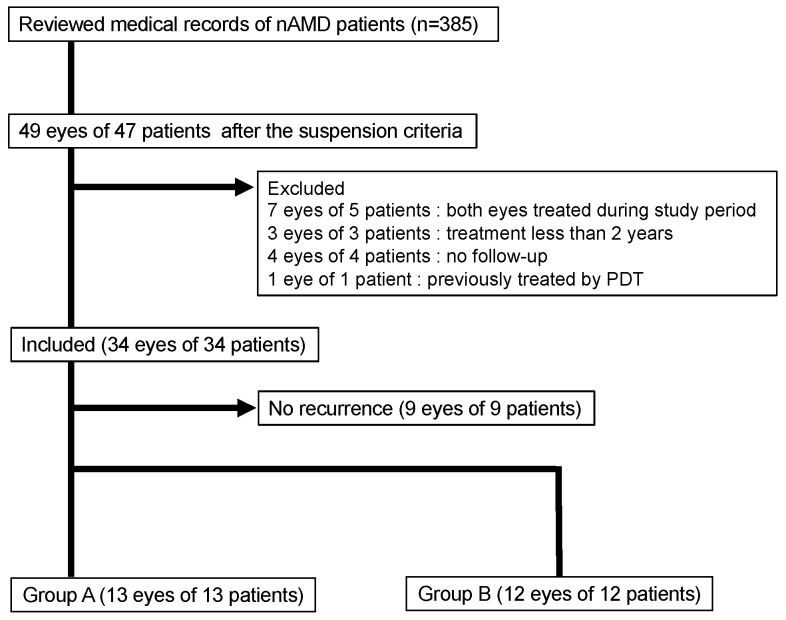
Flow chart showing the selection of participants. nAMD, neovascular age-related macular degeneration PDT, photodynamic therapy.

Of the 385 patients whose medical records were examined, there were 34 eyes of 34 patients who met the inclusion criteria. These included 12 women and 22 men, whose median age was 76 years, with a range of 72 to 82 years. A recurrence occurred in 25 of the 34 patients (73.5%) within the 5-year study period (Figure 1), and 13 patients were placed in Group A and 12 patients in Group B (Figure 1).

### 3.2. Recurrence Rate

**Figure 2:**
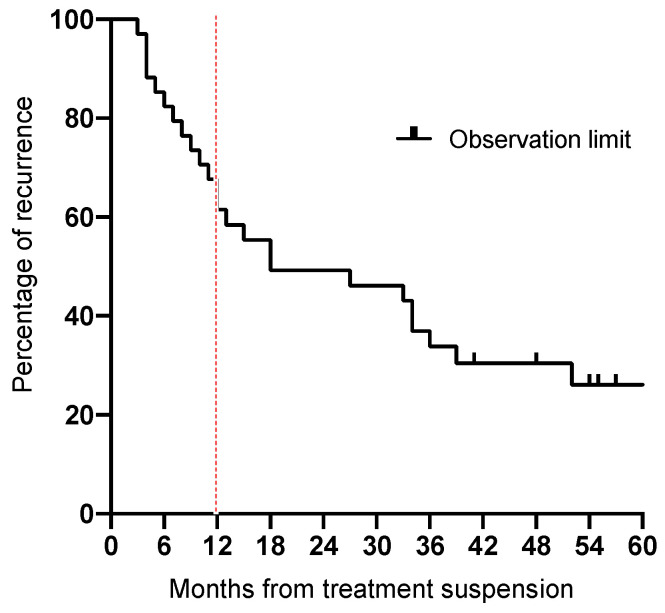
Kaplan–Meier survival plot showing the time and proportion of recurrences that occurred after the treatment suspension.

Thirteen (52.0%) of the twenty-five cases had a recurrence between 0 and 1 years, 4 (16.0%) between 1 and 2 years, 4 (16.0%) between 2 and 3 years, 2 (8%) between 3 and 4 years, and 2 (8%) between 4 and 5 years. The median time of recurrence after suspension was 12 months (Figure 2).

### 3.3. Clinical Characteristics of Patients

The differences in the baseline background factors of Group A and Group B were not significant (Table 1), and the correlations between the baseline background factors and the recurrence rate were also not significant (Table 2).

### 3.4. Visual Acuity Changes

**Figure 3:**
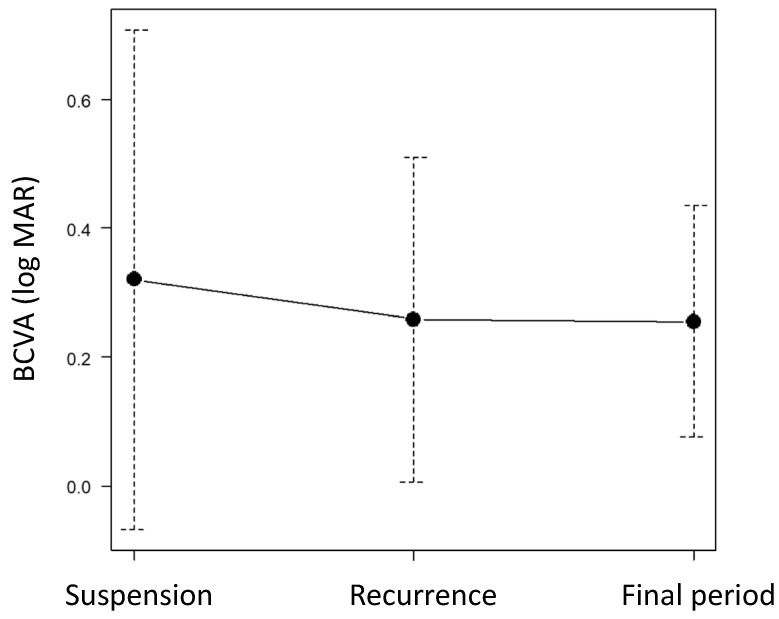
Visual acuity changes during the observation period of Group A.

The BCVA after the resumption of treatment in Group A was not significantly different from that at the suspension examination (*p* = 0.85, baseline) or at the final examination (*p* = 0.84) (Figure 3).

## 4. Discussion

The results showed that the rate of recurrence was 73.5% within 5 years after the suspension of treatment. The visual acuity at 5 years after the treatment suspension in Group A was not significantly different from that at the baseline.

We have reported that the recurrence rate after meeting the criteria for suspension of treatment was 50.0% within two years [17]. Nguyen et al. reported that the rate of recurrence of exudations after treatment suspension was 79% after 5 years, which is comparable to our findings [16]. It is known that the treatment suspension criteria can affect recurrence rates [16]. The criteria for suspension in the previous report were three injections at 12-week intervals and stable disease activity [16]. The suspension criteria used in this study were to continue anti-VEGF treatment every 12–16 weeks and to suspend the injections when the disease activity was stable for at least 48 weeks [17]. The criteria for suspending treatment in this study differed from those of earlier studies [16]. The differences were in the length of the dosing interval and its duration. The suspension criteria in the current study had a slightly longer dosing interval and a 12-week longer duration of continuous treatment. These differences suggested that the patients had stabilized disease activity before the treatment was suspended. The importance of allowing disease activity to stabilize before the suspension has been reported, and a past report has recommended extending the interval to 16 weeks before the suspension to reduce the disease activity [14]. Therefore, the fact that the recurrence rates in the two studies were comparable indicated that eyes that had been stable without exudation for a long period of time and the anti-VEGF therapy may need to be reinstituted after treatment suspension. This means that careful observations are needed for a long period of time after a suspension.

The factors associated with a recurrence of exudations have been reported. In one study that followed the patients for 5 years after the suspension, a treatment duration of less than three years was considered a high risk of recurrence [14]. According to a report examining the one-year follow-up after a suspension, the number of treatments during the 6 months prior to suspension was cited as a risk for recurrence. The factors that lowered the risk of recurrence included the longer duration of treatment and the visual acuity prior to the suspension [15]. We examined factors affecting the time of recurrence after suspension, but no significant factors were found, including the disease type, age, duration of treatment, and the last injection interval. Taken together, these results and those of previous studies suggest that the duration of treatments is related to the presence or absence of recurrences, but not to the interval of the recurrences.

By examining the changes in visual acuity after suspension of treatment in Group A, a new finding was made that early resumption of treatment after a recurrence may help maintain the visual acuity at the baseline value. This means that treatment should be initiated as soon as possible after a recurrence, as the visual acuity may not improve if the time for restarting treatment is prolonged. The importance of the timing of therapeutic interventions has been reported in the past. The results of the View study showed that between 52 and 64 weeks, patients with visual acuity loss of five or more letters in two consecutive visits were treated with reactive therapy at the second visit. The visual acuity trends in the patients were examined, but no subsequent improvement in visual acuity was obtained [18].

Although there have been reports of a decrease in visual acuity after a recurrence [16,18], the results of this study differed. The reason for the difference may be that the patients restarted treatment soon after the recurrence, even if the recurrence occurred within a year. Monthly follow-ups with OCT images allowed for early detection and restart of the treatments in case of a recurrence after discontinuation. The results of the current study indicate that even patients who had a recurrence within one year may be treated, as well as those who relapse after a long period of time, if treatment is resumed early. It is also important to repeat the examinations at short intervals, restart treatment immediately after a recurrence, and resume treatment as soon as possible.

The results of this study indicate that clinicians need to follow nAMD patients for a long time if treatment is interrupted. This is because a recurrence can occur after a discontinuation of treatment, as in Group B. If a recurrence is found, the clinician should follow the patient with caution, as immediate resumption of treatment can preserve visual acuity. Thus, we believe that it is important to continue the post-suspension follow-up protocol used in our study for a long period of time. The follow-up in our study is a frequent method using OCT, also every 1–2 months during the first year after the suspension and every 2–4 months during the second year.

## 5. Limitations

There are several limitations to this study. The first limitation is that this study was retrospective and had a small sample size. The reason for the small sample size was the small proportion of patients who met the discontinuation of treatment criteria. Munk et al. reported that about 15% of patients met the discontinuation criteria [19]. In addition, a large proportion of patients requested to be referred to a local doctor due to the burden of hospital visits after discontinuation of treatment. Our results showed that the clinical characteristics of Group A and Group B did not differ significantly, but only Group A had cases with pigment epithelial detachment (PED). PED has been reported to be a factor affecting recurrences and should be re-examined with an increased sample size [20]. The results of the hazard ratios for the correlations between the baseline background factors and the recurrence rate may change as the number of cases increases, as may the results for sex, subtypes of nAMD, PED, and anti-VEGF agents used.

Another limitation was that the suspension criteria were not a fixed number of weeks. The reason for this was that this study was retrospective and that suspension criteria extending to 16 weeks were proposed as recently as 2021 [13]. Another suspension criteria has also been used with patients with three visits 12 weeks apart with a dry retina [15]. Further studies are needed to unify the suspension criteria in the future.

## 6. Conclusions

We evaluated the recurrence rates of nAMD during a 5-year follow-up period after a suspension of anti-VEGF therapy. Our results indicated that the recurrence rate was highest within one year after suspension, with a smaller number of cases relapsing one year after the suspension. The results also indicated the possibility of long-term visual acuity preservation by immediately restarting treatment if the patient has a recurrence after the suspension of treatment. Clinicians should remember that the disease may recur several years after suspension.

## Figures and Tables

**Table 1:** Baseline characteristics of patients in Group A and Group B.

	Group A	Group B	*p* Value
Sex (Male:Female)	13 (10:3)	12 (8:4)	0.67 ^+^
Age (Median	80 [74, 83]	73.5 [71.25, 76.75]	0.15 *
Subtype (Type1:Type2:PCV)	4:2:7	7:2:3	0.22 ^+^
Treatment period (Median)	46 [38, 71]	43 [24, 60.25]	0.56 *
Treatment interval 12–14: 16 weeks	11:2	8:4	0.38 ^+^
GLD (μm)	2011 [804, 2847]	1406.5 [729, 1952.5]	0.21 *
Number of injections	13 [12, 19]	12 [12, 16.5 ]	0.58 *
PED	2	0	0.48 ^+^
Anti-VEGF drugs (ranibizumab/aflibercept)	2/11	5/7	0.20 ^+^

PED, pigment epithelial detachment; PCV, polypoidal choroidal vasculopathy; VEGF, vascular endothelial growth factor; GLD, greatest linear dimension. ^+^ Fisher’s exact test; * Mann–Whitney’s U test.

**Table 2:** Cox proportional hazard model for all the factors.

	Hazard Ratio	*p* Value	Lower 95% CI	Upper 95% CI
Age	1.113	0.277	0.917	1.349
Sex	0.422	0.518	0.031	5.756
Subtype	0.825	0.824	0.151	4.507
Treatment period(months)	0.985	0.56	0.935	1.037
Anti-VEGF drugs	1.036	0.97	0.097	11.01

CI: confidential interval.

## Data Availability

The datasets used during the current study are available from the corresponding author request.
